# Supplementary material for: Genome-Wide Identification of WRKY Transcription Factor Family in Chinese Rose and Response to Drought, Heat, and Salt Stress
Source: Genes (Basel). 2024 Jun 18;15(6):800. doi: 10.3390/genes15060800 (PMC11203230; doi:10.3390/genes15060800)
Supplement: Supplementary file 1 [file genes-15-00800-s001.zip › genes-3008848-supplementary.pdf]

Table S1 Characteristics of identified *RcWRKY* gene gamily

Gene	ID	Length	MW	pI	Instability Index	Aliphatic Index	GRAVY	Predicted Subcellular Location
RcWRKY57	PRQ28007	156	17862.44	5.11	45.44	44.23	-1.151	Nucleus
RcWRKY20	PRQ52554	476	52132.97	6.82	41.69	66.68	-0.893	Nucleus
RcWRKY16	PRQ49066	246	27951.42	9.08	52.77	63.78	-0.729	Nucleus
RcWRKY30	PRQ43243	292	32952.66	5.19	59.72	61.1	-0.801	Nucleus
RcWRKY32	PRQ45028	434	47596.44	6.3	46.61	70	-0.539	Nucleus
RcWRKY22	PRQ53481	534	58158.94	8.96	60.89	60.11	-0.761	Nucleus
RcWRKY4	PRQ58297	226	25453.83	9	37.17	68.58	-0.735	Peroxisomal
RcWRKY59	PRQ16657	897	101063.64	5.88	52.1	78.39	-0.547	Nucleus
RcWRKY48	PRQ34683	623	68204.85	7.28	51.71	43.71	-1.026	Nucleus
RcWRKY24	PRQ54294	201	22400.7	8.94	32.8	46.57	-0.942	Nucleus
RcWRKY5	PRQ58416	350	38922.46	5.9	45.02	62.94	-0.628	Nucleus
RcWRKY62	PRQ17584	223	25538.09	9.29	68.93	62.51	-0.764	Nucleus
RcWRKY49	PRQ34920	716	77262.71	5.63	52.27	50.15	-0.791	Nucleus
RcWRKY15	PRQ49015	120	13629.17	9.35	43.2	48.67	-1.026	Cytoplasm
RcWRKY14	PRQ48045	314	34188.83	9.56	49.83	66.5	-0.478	Nucleus
RcWRKY44	PRQ29826	496	53634.93	5.95	50.89	51.77	-0.768	Nucleus
RcWRKY42	PRQ29222	277	30212.29	5.34	62	50.32	-0.784	Nucleus
RcWRKY11	PRQ60203	426	45830.47	5.28	55.13	55.66	-0.669	Nucleus
RcWRKY35	PRQ37090	305	33158.47	9.85	51.11	57.87	-0.655	Nucleus
RcWRKY29	PRQ42789	506	55933.05	7.67	60.23	45.32	-0.964	Nucleus

RcWRKY25	PRQ41534	729	80160.02	5.81	54.43	63.81	-0.688	Nucleus
RcWRKY9	PRQ59650	166	19261.46	9.57	53.99	39.94	-1.207	Nucleus
RcWRKY2	PRQ57591	756	85116.83	7.89	43.57	59.68	-0.808	Nucleus
RcWRKY37	PRQ39862	191	21680.23	9.43	43.1	48.48	-0.859	Nucleus
RcWRKY3	PRQ58289	329	36121	7.7	53.66	48.33	-0.82	Nucleus
RcWRKY61	PRQ17455	359	40745.15	5.39	54.5	58.16	-0.782	Nucleus
RcWRKY21	PRQ52747	604	65496.99	5.28	45.45	58.38	-0.789	Nucleus
RcWRKY50	PRQ35468	1574	178605.88	5.66	47.38	92.12	-0.21	Cytoplasm
RcWRKY64	PRQ19998	457	50410.72	8.9	45.48	56.65	-0.839	Nucleus
RcWRKY34	PRQ45163	320	35696.02	7.04	54.2	51.84	-0.788	Nucleus
RcWRKY23	PRQ53664	490	53123.25	7.97	55.59	64.55	-0.619	Nucleus
RcWRKY17	PRQ50230	246	27179.2	4.97	53.33	58.21	-0.685	Nucleus
RcWRKY63	PRQ18143	292	32730.16	9.41	56.89	68.39	-0.658	Nucleus
RcWRKY58	PRQ16120	643	69796.83	6.69	50.59	57.12	-0.816	Nucleus
RcWRKY56	PRQ27739	322	34939.65	9.51	56.41	61.52	-0.469	Nucleus
RcWRKY55	PRQ27418	339	36616.51	9.53	51.17	60.21	-0.524	Nucleus
RcWRKY52	PRQ25953	350	39325.4	9.66	56.75	66.06	-0.819	Nucleus
RcWRKY39	PRQ40711	516	56371.85	6.84	57.61	47.07	-0.975	Nucleus
RcWRKY38	PRQ40678	322	35165.59	6.49	65.91	48.7	-0.878	Nucleus
RcWRKY40	PRQ40836	555	60358.93	7.68	46.65	60.88	-0.647	Nucleus
RcWRKY10	PRQ59963	1349	153304.2	6.55	48.25	90.25	-0.291	Cytoplasm
RcWRKY6	PRQ59641	348	38669.85	6.18	49.16	58.05	-0.751	Nucleus

RcWRKY65	PRQ20869	1415	159802.68	6.24	51.02	85.2	-0.295	Cytoplasm
RcWRKY7	PRQ59642	350	38755.93	6.02	44.27	54.83	-0.788	Nucleus
RcWRKY8	PRQ59647	1355	153169.09	5.93	44.14	88.55	-0.244	Cytoplasm
RcWRKY41	PRQ28392	583	62969.52	6.81	51.29	57.8	-0.773	Nucleus
RcWRKY60	PRQ16950	166	18921.29	9.4	43.94	59.22	-0.849	Nucleus
RcWRKY18	PRQ50421	324	35931.14	8.84	46.47	62.01	-0.856	Nucleus
RcWRKY33	PRQ45030	447	49490.84	6.44	45.08	68.64	-0.526	Nucleus
RcWRKY54	PRQ26893	391	43192.43	6.23	50.49	70.82	-0.612	Nucleus
RcWRKY13	PRQ46124	532	58532.74	5.56	62.37	61.41	-0.875	Nucleus
RcWRKY53	PRQ26892	305	33770.49	9.18	56.94	56.95	-0.837	Nucleus
RcWRKY45	PRQ31927	542	59233.07	6.3	43.55	67.01	-0.586	Nucleus
RcWRKY26	PRQ41792	314	34928.9	10	59.98	72.93	-0.653	Cytoplasm
RcWRKY19	PRQ52085	370	41584.86	6.83	53.14	55.65	-0.961	Nucleus
RcWRKY43	PRQ29367	353	38873.76	6.83	58.9	46.4	-0.897	Nucleus
RcWRKY67	PRQ21602	276	31621.61	4.87	66.77	51.16	-1.19	Nucleus
RcWRKY31	PRQ43414	478	52056.88	8.93	48.76	57.07	-0.842	Nucleus
RcWRKY28	PRQ42688	328	37628.5	5.49	50.48	60.03	-0.805	Nucleus
RcWRKY27	PRQ42687	373	42194.98	5.86	54.97	64.8	-0.647	Nucleus
RcWRKY36	PRQ39491	388	42399.34	6.23	57.39	47.81	-0.934	Nucleus
RcWRKY66	PRQ21005	78	9037.28	5.26	50.8	83.59	-0.465	Nucleus
RcWRKY12	PRQ60342	366	40135.86	6.55	51.26	66.39	-0.576	Nucleus
RcWRKY68	PRQ21604	142	16327.84	6.22	41.16	32.96	-1.613	Nucleus

RcWRKY47	PRQ32099	126	13962.85	7.84	58.84	76.43	-0.407	Cytoplasm
RcWRKY46	PRQ32097	581	63335.89	6.5	56.75	52.65	-0.854	Nucleus
RcWRKY1	PRQ57418	297	34069.93	9.31	42.75	60.3	-0.821	Nucleus
RcWRKY51	PRQ35800	140	15720.7	5.16	38.06	38.29	-1.329	Nucleus

Figure S1. Chromosomal localization of *RcWRKY* gene family members in Chinese rose. The scale on the left is in megabytes blue to yellow in chromosome indicates gene density. The chromosome number is displayed on the left side of the vertical bar; The right side of the vertical bar shows the gene location

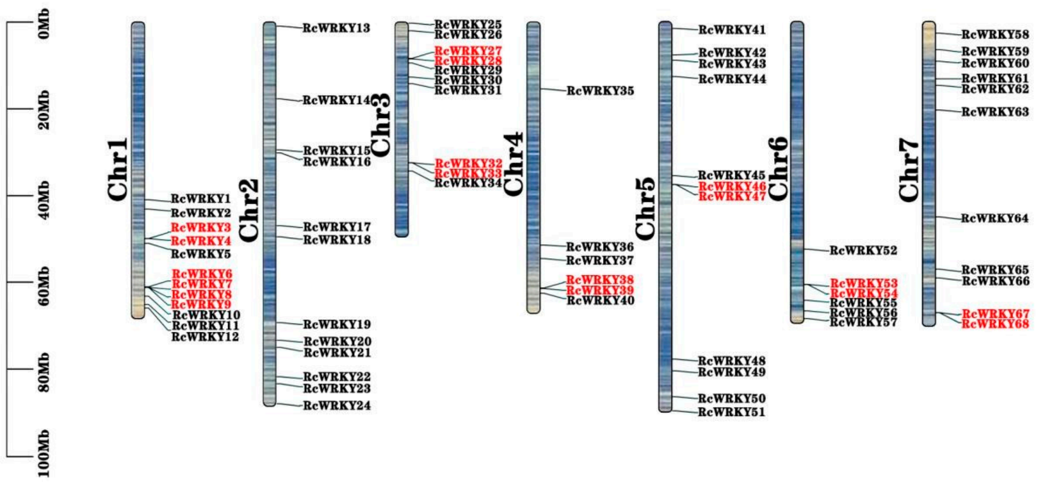

Table S2. Primers of *RcWRKY* gene needed for fluorescence quantification

Primer name	Sequence(5’-3’)	Annealing Temperature（℃）
Rcactin-qF	CCCCACTCAATCCCAAGGCAA	55.5
Rcactin-qR	CGGAAGTCCAGCACAATACCA	
RcWRKY5-qF	GTAGAGCCAAGAAGCGATAG	52.1
RcWRKY5-qR	TACTGTGAATGTGAAGCCG	
RcWRKY14-qF	CTGCCCTTCACTGCTCC	53.3
RcWRKY14-qR	TAGTCCCCTCCATTCCG	
RcWRKY16-qF	AGCAGGGCTTGTTTGATA	51
RcWRKY16-qR	ATAGGCTTTGAGGGATTGT	
RcWRKY28-qF	GTTCACAAACACCCTTCGTATC	56.3
RcWRKY28-qR	CAGCATCATCAAGAGCATCC	
RcWRKY39-qF	AGTGAGGAAGTTGGAGATGC	53.1
RcWRKY39-qR	CACAGTCCGATGAGAAGAAG	
RcWRKY49-qF	AACCCACCAAAGTTCAAAATCA	58.3
RcWRKY49-qR	CTGCCACCAACAGTATCAAAAG	
RcWRKY56-qF	AGCAATCCCAATCCTCA	50.7
RcWRKY56-qR	CCGTCCCCAGACATAAA	
RcWRKY62-qF	CCAACCATTCCTATCGCCTC	60.5
RcWRKY62-qR	CGCCACGCCCACACATC	
